# Intermarriage or the Mode in Which, and the Causes Why, Beauty, Health and Intellect, Result from Certain Unions, and Deformity, Insanity and Disease from Others

**Published:** 1852-01

**Authors:** 


### ARTICLE XII.

*Intermarriage or the mode in which, and the causes why, Beauty, Health and Intellect, result from certain unions, and Deformity, Disease and Insanity from others: demonstrated by delineations of the structure and forms, and description of the functions and capacities, which each parent, in every pair, bestows on Children,---in conformity with certain natural laws, and by an account of corresponding effects in the breeding of animals. With eight illustrative drawings.* By ALEXANDER WALKER. Philadelphia: Lindsay & Blakiston, 1851. (From the Publishers through S. C. Griggs & Co.)

This work treats of one of the most important subjects to the welfare of posterity that can claim the attention of the young man or woman when about forming the matrimonial connection.

There can be no doubt of the great influence of parentage upon posterity, in fact, almost every thing in reference to the physical and mental characteristics of progeny may be pre dicted from a knowledge of the parents.

The subject though but recently reduced to a science should claim an important consideration in the education of youth, for among the means of elevating our species, in the scale of physical, intellectual and moral development, there is no one that exerts a more powerful influence than that of a proper adaptation of parents for the production of an improved progeny. Our readers will better understand the scope of the work from the following extract from the advertisement at the commencement of the volume:

"The great object of this work is altogether new and heretofore unattempted—the establishment not merely of a new science-- but of that science which is by far the most interesting to humanity--the science which, for the first time, points out and explains all the natural laws that, according to each particular choice in intermarriage, determine the precise forms and qualities of the progeny,---which unfolds the mode in which, and the causes why beauty, health and intellect result from certain unions, and deformity, disease and insanity from others,---and which enables us, under all given conditions, and with absolute certainty, to predict the degree and kind of these, which must result from each intermarriage.

The philosophical bases of this science have, moreover, nothing to do with hypothesis or supposition ;---they are the indisputable, though hitherto unapplied, facts of anatomy and physiology ;---and their present popular applications are rendered subjects of absolute demonstration by descriptions and drawings of families (some of them well known to the public ;) while every reader has the power to add to their number among the families of his acquaintance. They are further subjected to demonstration by all the more important facts here stated, as to the breeding of domesticated animals—facts which have not hitherto been explained or understood, and consequently have not hitherto afforded those principles on which the breeder may *now* act, with perfect certainty of the desired result.

In the First Part of the work is given an account of the physiological conditions connected with and terminating in Love, --the period of puberty, and the remarkable and interesting changes which it causes in the locomotive system and the voice, in the vital or nutritive system, and in the mental or thinking system, especially of woman. This is rendered altogether popular.

In the Second Part are described the sexual relations arising from these conditions, and connected with or leading to Intermarriage,—useful guidance and dangerous restraint, unnatural indulgence and absolute continence, and the necessity of intermarriage---subjects entirely popular and deeply interesting to both sexes.

In the Third Part are described the circumstances resulting from the preceding relations, and connected with or productive of Progeny,—the natural preference for the various kinds of beauty for the first time explained, the state of marriage, and the propogation of forms and qualities.

In the Fourth Part are enunciated the newly discovered laws regulating the Resemblance of Progeny to Parents, the law of selection where both parents are of the same variety, the law of crossing where each parent is of a different variety, the law of in-and-in breeding where both parents are of the same family, the law of sex, and the law of maternal nutrition (none of them heretofore observed, and all of them here physiologically demonstrated,) as well as the eircumstances modifying these laws, and the consequent easy improvement of families in beauty of forms and excellence of functions.

In the Fifth and Sixth Parts are described the vague methods of regulating progeny adopted in the breeding of Domesticated Animals,—in in-and-in, selection and crossing, and the application of the natural laws to the breeding of these animals—horses, cattle and sheep.

In the Seventh and Eighth Parts are described the vague methods of effecting progeny among Mankind,—in in-and-in, selection and crossing, and the transcendently important subject of choice in intermarriage, as prescribed by the natural laws, and as calculated to correct each particular defect of the locomotive, the vital or nutritive, and the mental or thinking system, that may exist in any family or any individual."

